# Polycystic ovary syndrome associated with increased adiposity interferes with serum levels of TNF-alpha and IL-6 differently from leptin and adiponectin

**DOI:** 10.20945/2359-3997000000197

**Published:** 2020-03-04

**Authors:** Nathália Sigilló Cardoso, Victor Barbosa Ribeiro, Sabrina Graziani Veloso Dutra, Rui Alberto Ferriani, Ada Clarice Gastaldi, João Eduardo de Araújo, Hugo Celso Dutra de Souza

**Affiliations:** 1 Departamento de Biomecânica, Medicina e Reabilitação do Aparelho Locomotor Faculdade de Medicina de Ribeirão Preto Universidade de São Paulo Ribeirão Preto SP Brasil Departamento de Biomecânica, Medicina e Reabilitação do Aparelho Locomotor, Faculdade de Medicina de Ribeirão Preto, Universidade de São Paulo (FMRP-USP), Ribeirão Preto, SP, Brasil; 2 Departamento de Ginecologia e Obstetrícia Faculdade de Medicina de Ribeirão Preto Universidade de São Paulo Ribeirão Preto SP Brasil Departamento de Ginecologia e Obstetrícia, Faculdade de Medicina de Ribeirão Preto, Universidade de São Paulo (FMRP-USP), Ribeirão Preto, SP, Brasil; 3 Instituto Federal de São Paulo Jacareí SP Brasil Instituto Federal de São Paulo, Jacareí, SP, Brasil

**Keywords:** Adipokines, polycystic ovary syndrome, body fat, metabolism

## Abstract

**Objective:**

The aim of this study was to investigate polycystic ovary syndrome (PCOS) and to explore the relationship between body fat percentage and metabolic markers.

**Subjects and methods:**

Sedentary women were assigned to PCOS (N = 60) and CONTROL (N = 60) groups. Each group was subdivided into three subgroups according to body fat percentage (22-27%, 27-32% and 32-37%). The protocol consisted of assessments of glucose, insulin, androgens, follicle stimulating hormone (FSH), luteinizing hormone (LH), 17-hydroxyprogesterone (17-OHP), leptin, adiponectin, tumor necrosis factor (TNF-α) and interleukin-6 (IL-6).

**Results:**

The PCOS subgroups showed higher concentrations of androgens, LH and 17-OHP. Leptin showed direct relationship with increased body fat percentage, whereas adiponectin showed the inverse effect. However, both were unaffected by PCOS. TNF-α and IL-6 were higher in PCOS women and showed a direct relationship with increased body fat percentage. Glucose showed direct relationship with body fat percentage, whereas insulin presented higher values in PCOS women and direct relationship with increased body fat percentage.

**Conclusions:**

Our findings indicate that PCOS and body fat percentage directly influence concentrations of insulin, TNF-α and IL-6, whereas leptin and adiponectin are influenced only by the increase in body fat percentage in these women. Arch Endocrinol Metab. 2020;64(1):4-10

## INTRODUCTION

Polycystic ovary syndrome (PCOS) is the most common endocrinopathy that occurs during reproductive life and it affects approximately 5% to 15% of women worldwide ( 1 ). The characteristics associated with PCOS, such as hirsutism, acne, ovaries with a polycystic pattern and increased body fat percentage are not consistent. Thus, the diagnosis is made based on at least two of the three Rotterdam consensus criteria: 1) menstrual irregularity (oligomenorrhea/amenorrhea), 2) clinical and/or laboratory hyperandrogenism, and 3) Standard ultrasound diagnosis of polycystic ovaries (presence of at least one ovarian follicle having 12 or more measuring from 2 to 9 mm in diameter, and/or total volume > 10 cm^3^) ( 2 ). Moreover, metabolic disorders are commonly associated with PCOS and include hyperinsulinemia, dyslipidemia and type II diabetes, which are associated with the development of cardiovascular disorders and disease ( 2 - 4 )

According to studies in the literature, women with PCOS may experience dysregulation in adipose tissue, in the synthesis of hormonal factors known as adipokines and gene expression profile of omental adipose tissue of women with PCOS demonstrates that genes in several classes are dysregulated ( 5 ). Some of these changes are characterized by increased serum concentrations of leptin, tumor necrosis factor (TNF)-α and interleukin (IL)-6 and by the reduced production of adiponectin ( 4 - 6 ). The relationship between PCOS and adipokines is controversial because this endocrine-metabolic disorder is often associated with an increased body fat percentage ( 7 ). However, there is controversy about adiponectin role in inflammation since its plasma concentration decreases in some inflammatory diseases, for example in obesity and PCOS, and increases under some other inflammatory conditions – rather than decreased – in classic chronic inflammatory/autoimmune diseases that are unrelated to increased adipose tissue, such as rheumatoid arthritis type 1 diabetes ( 8 ).

The aim of this study was to investigate whether the changes observed in the serum levels of leptin, adiponectin, TNF-α e IL-6 in women with PCOS resulted from increased body fat or were the main result of endocrinopathy. Thus, we evaluated the relationship among three body fat percentage levels (22%-27%, 27%-32% and 32%-37%) and androgenic and metabolic lipid profiles, with an emphasis on adipokines, by analyzing the concentrations of leptin, adiponectin, TNF-α and IL-6.

## SUBJECTS AND METHODS

### Sampling

We studied 120 sedentary women, who did not perform regular exercise (aged 33 ± 3 years), and were screened at the outpatient clinic of Gynecology and Obstetrics, Clinical Hospital of the Ribeirão Preto Medical School, University of São Paulo (HCFMRP/USP). The women were assigned to one of two groups: PCOS group (N = 60) according to the Rotterdam consensus and CONTROL group (N = 60) without PCOS. They were at the clinic for routine gynecological exams. All volunteers with PCOS selected for the study had polycystic ovaries, 78,3% had biochemical hyperandrogenism, 73,3% had clinical hyperandrogenism and 90% had irregular menstrual cycles.

Each group was subdivided into three smaller subgroups (n = 20) according to body fat percentage (22%-27%, 27%-32% and 32%-37%) ( 9 ). The women were randomly selected and evaluated for percentage of fat, and subsequently included in the study to complete the number of 20 volunteers per subgroup.

The exclusion criteria for the study were women younger than 18 or older than 40 years, the use of contraceptives, pharmacological treatment, smoking, drinking, cognitive disorders that would hinder the understanding and execution of tests, disabling musculoskeletal and cardiovascular diseases, Cushing’s syndrome, congenital adrenal hyperplasia, androgen-secreting neoplasm and any form of endocrine disorders.

All of the women were informed of the study’s objectives and provided their consent to participate in the study. The study was approved by the Research Ethics Committee of the HCFMRP/USP, São Paulo, Brazil (Process n^o^11613/2008).

### Laboratory tests

Blood samples were collected between 7 and 10 hours, on an empty stomach, in the follicular period of the cycle (2nd-7th day of menstruation) or during amenorrhea, using vacutainer tubes (BD-Becton Dickinson, Plymouth, UK). After blood collection, the samples were centrifuged for 10 min at 4 °C and 2.500 rpm. The plasma isolated from each patient was stored in Eppendorf tubes (1.5-mL), which were immediately placed in a freezer at -80 °C for subsequent analysis. The only exception was serum glucose, which was analyzed immediately after obtaining the blood samples using the Hexokinase-UV method. The volunteers were instructed to fast for 12 hours, to avoid physical activities and beverages containing alcohol or caffeine 48 hours before the blood sampling. Serum levels of triglycerides (TG) and cholesterol (LDL, low-density lipoprotein; HDL, high-density lipoprotein) were measured using an enzymatic spectrophotometer (DU-640; Beckman, Fullerton, CA). Specific radioimmunoassay procedures (Siemens Medical Diagnostics, Los Angeles, CA, USA) were conducted to quantify the plasma levels of leptin, total adiponectin, testosterone, androstenedione, 17-hydroxyprogesterone (17-OHP), luteinizing hormone (LH) and follicle stimulating hormone (FSH). IL-6 and TNF-α concentrations were determined using chemiluminescence immunoassays. Insulin resistance was assessed using HOMA-IR = [fasting glucose (mg/dL) × 0.05551] × [fasting insulin (μU/mL)]/22.5, and considered resistance when HOMA-IR > 2.71 ( 10 ).

### Body composition and body fat percentage

Anthropometric measurements including body mass index (BMI), waist circumference, hip circumference and waist/hip ratio were taken. BMI was obtained using the formula W/H^2^, where W was the weight of the women in kilograms and H was the height of the women in meters. Body composition and body fat percentage were evaluated using the bioelectrical impedance method (BIA 310, Biodynamics Corporation, Shoreline, WA).

### Statistical analysis

All of the statistical tests were performed using SigmaStat 3.5 software (Systat Software Inc., San Jose, CA, USA). The results are shown as the mean ± SEM (standard error of the mean). The effects of PCOS and body fat percentage were assessed using two-way analysis of variance (ANOVA). When appropriate, post hoc comparisons were performed using the Student Newman-Keuls test. Differences were considered to be statistically significant when p < 0.05.

## RESULTS

As seen on Table 1 , the body composition data show no significant differences in age and height between CONTROL and PCOS groups (intergroup comparison). Also there were no significant differences in body weight, body mass index (BMI), waist/hip ratio and average percentage of body fat. However, when these parameters were compared intragroup (22%-27%; 27%-32%; and 32%-37%), we observed significant increase related to the range of the percentage of body fat.

**Table 1:** Anthropometric values of women from the CONTROL and PCOS groups subdivided according to body fat percentage

Anthropometric values	22%-27%		27%-32%		32%-37%	
	CONTROL (20)	PCOS (20)	CONTROL (20)	PCOS (20)	CONTROL (20)	PCOS (20)
Age (years)	32 ± 7	34 ± 5	33 ± 7	35 ± 7	31 ± 4	33 ± 7
Height (cm)	165 ± 7	162 ± 7	166 ± 7	165 ± 5	163 ± 5	161 ± 6
Weight (kg)	63 ± 6	59 ± 4	72 ± 7^a^	69 ± 6^d^	79 ± 11^a,b^	76 ± 9^d, e^
BMI, kg/m^2^	23,1 ± 2,6	22,6 ± 2,9	26,3 ± 3,0^a^	25,5 ± 3,0^d^	29.8 ± 4.3^a,b^	29,9 ± 4,5^d,e^
WHR	0.75 ± 0,02	0.74 ± 0,01	0.79 ± 0,02^a^	0.81 ± 0,02^d^	0.84 ± 0,01^a,b^	0.83 ± 0,02^d,e^
Body fat, %	23,6 ± 1,7	24,4 ± 2,1	29.8 ± 3.2^a^	30,3± 1,5^d^	35.7 ± 3.2^a,b^	35,2 ± 1,5^d,e^

Data is expressed as the mean ± SEM values. PCOS, polycystic ovary syndrome; BMI, body mass index; WHR, waist-to-hip ratio.^a^P < 0.05 vs. CONTROL (22%-27%); ^b^P < 0.05 vs. CONTROL (27%-32%); ^d^P < 0.05 vs. PCOS (22%-27%); and ^e^P < 0.05 vs. PCOS (27%-32%).

The metabolic parameter analysis illustrated in Table 2 show no differences in lipoproteins intra (adiposity) and intergroup (CONTROL *vs.* PCOS). Triglycerides analyses showed differences related to adiposity within each group. The CONTROL subgroup with the greater body fat percentage (32%-37%) showed increased triglyceride values when compared to the other subgroups (22%-27% e 27%-32%). On the other hand, comparisons within the PCOS group showed that the triglycerides increased directly proportional to the increase in the percentage of body fat.

**Table 2:** Metabolic parameters of women from the CONTROL and PCOS groups subdivided according to body fat percentage

Metabolic parameters	22%-27%		27%-32%		32%-37%	
	CONTROL (20)	PCOS (20)	CONTROL (20)	PCOS (20)	CONTROL (20)	PCOS (20)
Cholesterol, mg/dL	196 ± 19	200 ± 24	212 ± 45	205 ± 32	209 ± 16	219 ± 32
HDL, mg/dL	45 ± 8	44 ± 8	42 ± 11	42 ± 5	43 ± 7	41 ± 6
LDL, mg/dL	107 ± 11	103 ± 16	107 ± 16	108 ± 17	114 ± 13	118 ± 25
TG, mg/dL	115 ± 21	109 ± 19	121 ± 34	140 ± 33^d^	149 ± 39^a,b^	165 ± 44^d,e^
FPG, mg/dL	86 ± 8	85 ± 12	88 ± 11	93 ± 12^d^	90 ± 10	95 ± 9^d^
FI, U/ml	9,14 ± 1,5	10,2 ± 2,8	9,6 ± 2,9	12.9 ± 2,8^b,d^	9,7 ± 2,1	14,3 ± 2,5^c,d^
HOMA – IR	1,94 ± 0,33	2,16 ± 0,77	2,09 ± 0,65	2,98 ± 0,77^b,d^	2,14 ± 0,41	3,35 ± 0,76^c,d^

Data is expressed as the mean ± SEM values. PCOS, polycystic ovary syndrome; HDL, high-density-lipoprotein-cholesterol; LDL, low-density-lipoprotein-cholesterol; TG, triglycerides; FPG, fasting plasma glucose; FI, fasting plasma insulin. ^a^P < 0.05 vs. CONTROL group (22%-27%); ^b^P < 0.05 vs. CONTROL group (27%-32%); ^c^P < 0.05 vs. CONTROL group (32%-37%); ^d^P < 0.05 vs. PCOS group (22%-27%); and ^e^P < 0.05 vs. PCOS (27%-32%).

Table 2 also illustrates the glycemia, insulin and HOMA-IR index values obtained in all groups. There were no differences within the CONTROL group. However, PCOS group showed several differences. PCOS subgroups with the greater body fat percentages (27%-32% and 32%-37%) showed increased values for glycemia, insulin and HOMA-IR index when compared to the PCOS subgroup with lower body fat percentage (22%-27%). While there were no intergroup (CONTROL *vs.* PCOS) differences in glycemia, fasting insulin and HOMA-IR index were higher for the PCOS subgroups with greater body fat percentages (27%-32% and 32%-37%) when compared to their respective CONTROL subgroups (27%-32% and 32%-37%).

As seen on Table 3 , intergroup (CONTROL *vs.* PCOS) analysis of androgens (testosterone and androstenedione) show higher values for the PCOS subgroups when compared to their respective CONTROL subgroups. On the other hand, intragroup (adiposity) analysis showed no differences within the CONTROL group, while the PCOS subgroups with the greater body fat percentages (27%-32% and 32%-37%), showed higher testosterone levels when compared to the lower body fat percentage subgroup (22%-27%). The PCOS subgroup with the greater body fat percentage (32%-37%) showed higher androstenedione levels when compared to the other PCOS subgroups (22%-27% and 27%-32%).

**Table 3:** Hormonal parameters of women from the CONTROL and PCOS groups subdivided according to body fat percentage

Hormonal parameters	22%-27%		27%-32%		32%-37%	
	CONTROL (20)	PCOS (20)	CONTROL (20)	PCOS (20)	CONTROL (20)	PCOS (20)
Testosterone, nmol/L	1,55 ± 0,69	2,51 ± 1,13^a^	1,59 ± 0,39	3,81 ± 1,69^b,d^	1,62 ± 0,88	4,08 ± 1, 62^c, d^
Androstenedione, nmol/L	4,22 ± 2,07	7,78 ± 2,95^a^	4,26 ± 1,48	8,92 ± 3,12^b^	4,45 ± 2,26	9,29 ± 3,32^c^
17-OHP (ng/mL)	0,86 ± 0,37	1,17 ± 0,36^a^	0,92 ± 0,45	1,21 ± 0,43^b^	0,91 ± 0,34	1,27 ± 0,48^c^
FSH (U/L)	6,63 ± 2,55	4,84 ± 1,47^a^	7,11 ± 2,17	5,11 ± 2,29^b^	6,81 ± 2,35	3,84 ± 1,12^c^
LH (U/L)	6,41 ± 2,46	7,91 ± 2,19	6,22 ± 1,66	8,41 ± 2,58^b^	6,74 ± 1,88	9,11 ± 1,45^c^
Leptin, ng/ml	16,51 ± 6,89	18,14 ± 7,94	22,61 ± 7,25^a^	24,71 ± 9,12^d^	33,49 ± 8,32^a, b^	33,05 ± 9,05^d, e^
Adiponectin, ng/mL	12,35 ± 5,38	11,81 ± 5,33	8,75 ± 5,92	8,72 ± 5,75	7,13 ± 4,83^a^	7,28 ± 4.38^d^
TNF-α, pg/mL	3,78 ± 2,91	4,25 ± 3,07	4,41 ± 2,40	5,36 ± 1,95^b^	5,68 ± 1,97^a, b^	6,86 ± 2,63^c, d, e^
IL-6, pg/mL	1,82 ± 1,77	2,04 ± 1,80	1,92 ± 1,58	3,03 ± 2,54	4,24 ± 2,68^a, b^	6,72 ± 3,46^c, d, e^

Date are expressed as the mean ± SEM values. PCOS, polycystic ovary syndrome; 17-OHP, 17-hydroxyprogesterone; FSH, follicle stimulating hormone; LH, luteinizing hormone; TNF-α, tumor necrosis factor-α; IL-6, interleukin-6. ^a^P < 0.05 vs. CONTROL group (22%-27%); ^b^P < 0.05 vs. CONTROL group (27%-32%); ^c^P < 0.05 vs. CONTROL group (32%-37%); ^d^P < 0.05 vs. PCOS group (22%-27%); and ^e^P < 0.05 vs. PCOS (27%-32%).

Intergroup analysis (CONTROL *vs.* PCOS) of 17-OHP and LH show higher values for the PCOS subgroups when compared to their respective CONTROL subgroups. On the other hand, intergroup analysis (CONTROL *vs.* PCOS) of FSH show lower values for the PCOS subgroups. Adipokines analysis show significant increase in leptin positively correlated with increase in body fat for both the CONTROL and the PCOS groups, while adiponectin showed the opposite. In this way it was possible to observe that PCOS patients (32%-37%) exhibited higher levels of testosterone and also lower levels of adiponectin than PCOS (22%-27%). Interestingly, some previous studies have shown *in vitro* ( 11 ), as well *in vivo* ( 12 ) that adiponectin can decrease androgen production in theca.

In addition, while intragroup comparisons (adiposity) within the CONTROL and the PCOS showed no differences in adiponectin for the subgroups with the greater body fat percentages (27%-32% and 32%-37%), these groups showed lower adiponectin levels when compared to the subgroups with lower body fat percentage (22%-27%). Intergroup comparisons (CONTROL *vs.* PCOS) showed no differences in leptin and adiponectin. Intragroup comparisons (adiposity) showed higher levels of TNF-α and IL-6 for the subgroup with greater body fat percentage (32%-37%) when compared to the other subgroups (22%-27% and 27%-32%). Intergroup comparisons (CONTROL *vs.* PCOS) showed higher levels of TNF-α and IL-6 for the subgroup with greater body fat percentage (32%-37%) when compared to the respective CONTROL subgroup.

## DISCUSSION

We have investigated the relationship between important metabolic parameters and body fat percentage in women with PCOS. Our results indicate that there are important differences among metabolic parameters evaluated in women with and without PCOS, especially when body fat was considered.

In our study, increased insulin resistance was found in PCOS group when compared to the CONTROL group, corroborating the data from previous studies ( 13 ). Obesity is often associated with insulin resistance and hyperinsulinemia. However, eutrophic individuals, may also exhibit these conditions, mainly due to visceral fat accumulation and metabolic and endocrine differences ( 13 - 15 ). The increased insulin resistance might be playing a central role in the development of endothelial dysfunction (early event in atherosclerosis process), increasing oxidative stress and secretion of hormones and adipokines by the adipose tissue ( 16 - 18 ). Endothelial dysfunction is also characterized by lower production of nitric oxide and its production not only modulates the tone of vascular smooth muscle, but also inhibits several atherogenic processes ( 16 - 19 ). These processes are the adhesion of platelets and monocytes, LDL oxidation, synthesis of inflammatory cytokines and proliferation of vascular smooth muscle cells ( 16 - 19 ). In addition, these results can influence hormonal regulation, since increased insulin resistance may interfere with the steroidogenesis by increasing the androgens synthesis ( 20 ).

Further, according to the literature, the increase of testosterone and other androgens may influence the mechanisms involved in the modulation of insulin receptors ( 21 ). These alterations contribute to increased insulin resistance and hyperinsulinemia which reveal a cascade and cyclic effect among the various components present in the syndrome ( 20 - 21 ). In this study, serum levels of testosterone and androstenedione were higher in PCOS women. In addition, testosterone levels were significantly elevated in the PCOS group with the highest fat percentage. This finding is consistent with previous results reported in literature and suggests that testosterone can induce a proliferative effect in pre-adipocytes ( 22 ).

It has also been suggested that androgenic hormones and insulin resistance interfere directly in adipokines concentrations, which are considered important metabolic and inflammatory markers ( 4 , 6 ). They are produced by adipose tissue and several of them are involved in the development of complications associated with increased body fat, for example, insulin resistance ( 4 ). Therefore, it is common to observe an increase in serum leptin, TNF-α and IL-6 levels accompanied by reduced levels of adiponectin ( 4 - 6 ). However, the association between these adipokines and PCOS is still controversial and there is no conclusion whether these changes in adipokines levels in PCOS women are correlated to increased body fat or endocrine disorder.

In our study, analysis of serum adiponectin and leptin showed no differences between PCOS and CONTROL groups, however, there was a direct relationship between these hormones and body fat percentage values. Adiponectin levels decreased with an increase in body fat percentage, confirming the results of other authors which also related to the presence of hyperandrogenism ( 4 , 6 ). The consequences of this decrease have been reported in other studies. It has been associated with progression of type II diabetes, endothelial dysfunction ( 23 ) and increased risk of cardiovascular disease in women ( 24 ). On the other hand, leptin levels had a positive correlation with body fat percentage, since their values were significantly higher when the percentage increased, which has been reported in other studies ( 4 , 6 - 7 ). However, no significant differences in leptin levels were detected intergroup.

Thus, despite the controversies in the literature, there is strong evidence from the present study as well as other studies, indicating that the increase in body fat percentage could be responsible for increased production of leptin and decreased adiponectin ( 25 ). While other studies have suggested that leptin production might be regulated by insulin action, higher body fat percentage has been shown to play a central role in leptin increase ( 8 , 25 ). The results in the present study corroborate this idea. Although, we observed an increase in fasting insulin and in HOMA-IR in PCOS women, their leptin levels were similar to the women in their CONTROL group.

However, the production of other adipokines may involve more than one mechanism. This statement is based on the findings of the present study. Such as leptin, the elevated concentrations of TNF-α and IL-6 in groups with higher fat body percentages (27%-32% and 32%-37%) suggest an effect of body fat. In turn, the PCOS group also had higher serum concentrations of TNF-α and IL-6 compared to the CONTROL group, suggesting in this case an effect of PCOS. Possible explanations suggest an association between hyperinsulinemia and the expression of these pro-inflammatory markers has been described ( 26 - 27 ).

In this regard, some studies have suggested that increased TNF-α concentrations may impair the translocation of GLUT4 receptors ( 28 - 29 ), while IL-6 may have pro-inflammatory properties that lead to insulin resistance ( 26 ). Increases in IL-6 and TNF-α were also observed, particularly in the PCOS insulin resistance group ( 18 ). In addition, TNF-α can reduce the activity of lipoprotein lipase ( 30 ) and IL-6 increase the uptake of fatty acids to adipose tissue and to the liver, which in turn promotes hypertriglyceridemia ( 31 ). On the other hand, the reverse situation also appears to be true, since it was observed that insulin and obesity stimulates the gene expression of TNF-α and IL-6 in adipose tissue ( 32 ). There is evidence that the increase of testosterone levels may be a trigger for producing these markers ( 33 ). These studies may help us explain why women with PCOS in our study displayed higher levels of TNF-α and IL-6 when compared to their counterparts. Additionally, PCOS patients show also increase in the generation of reactive oxygen species by mononuclear cells which are located in the stromal vascular compartment of visceral adipose tissue. This increase happens in response to their hyperglycemia and it might play an important role in the development of hyperandrogenism and insulin resistance through the activation of NF-κB and increased transcription of the TNF-α gene. This characterizes the PCOS as a proinflammatory state ( 34 ).

Finally, our study shows that there are differences in the behavior of adipokines in women with PCOS; however, the increase in percentage of body fat is an important factor for these changes. Thus, decreased body fat shows lower expression of inflammatory markers, elucidating the hypothesis that the expression of these markers is therefore primarily due to excess body fat, as observed in groups with lower fat percentage (22%-27%). However, TNF-α and IL-6, in addition to the increase in the percentage of body fat, were also found to be higher in the PCOS group when compared to the control when the fat percentage was 32%-37% and the TNF-α higher in the PCOS group in relation to the control also in the percentage 27%-32%.

In general, our study presented important and very representative results. However, there is limitation. To evaluate body fat, we use bioimpedance equipment. For future studies that need to perform the same stratification we suggest the use of Dual X-ray absorptiometry (DXA) that has been referenced as reference standard ( 35 ).

We conclude that serum levels of leptin and adiponectin are altered in women with intermediate and high degrees of adiposity and is independent of the presence of PCOS, showing that the degree of alteration in the serum levels of these adipocytokines is proportional to the degrees of adiposity. Differently, serum levels of TNF-α and IL-6 are increased only when the level of adiposity is very high, and are even higher in the presence of PCOS, suggesting that the role of obesity in the etiology of PCOS is mainly related to higher secretion of TNF-α and IL-6 adipocytokines by adipocytes, being little influenced by leptin and adiponectin in this population.
